# Survival benefits of adjuvant chemotherapy after conversion surgery in patients with advanced pancreatic cancer

**DOI:** 10.3389/fonc.2024.1510016

**Published:** 2025-01-07

**Authors:** Yoon Suk Lee, Jang Won Lee, Hak Jun Kim, Jung Won Chun, Jong-Chan Lee, Dong Kee Jang, Jin-Hyeok Hwang, Young Ae Kim, Sang Myung Woo

**Affiliations:** ^1^ Department of Internal Medicine, Ilsan Paik Hospital, Inje University College of Medicine, Goyang, Republic of Korea; ^2^ Division of Cancer Control & Policy, National Cancer Control Institute, National Cancer Center, Goyang, Republic of Korea; ^3^ Department of Artificial Intelligence Convergence, Hallym University Graduate School, Chuncheon, Republic of Korea; ^4^ Research Institute, Center for Liver and Pancreatobiliary Cancer, National Cancer Center, Goyang, Republic of Korea; ^5^ Department of Internal Medicine, Seoul National University Bundang Hospital, Seoul National University College of Medicine, Seongnam, Republic of Korea; ^6^ Department of Internal Medicine, Seoul National University Boramae Medical Center, Seoul, Republic of Korea

**Keywords:** surgical procedures, operative, FOLFIRINOX, albumin-bounded paclitaxel, carcinoma, pancreatic ductal, survival analysis

## Abstract

**Background:**

Oncologic outcomes of conversion surgery for advanced pancreatic cancer (PC) have scarcely been reported. Therefore, this study aimed to investigate the outcomes of conversion surgery with preoperative treatment of FOLFIRINOX or gemcitabine with nab-paclitaxel (GnP) for patients with advanced PC including locally advanced or metastatic PC.

**Methods:**

Using the National Health Insurance database between 2005 and 2020, we identified patients who underwent conversion surgery after chemotherapy with FOLFIRINOX or GnP for advanced PC. The patients were categorized based on preoperative treatment. Survival outcomes were evaluated based on the date of cancer diagnosis and conversion surgery.

**Results:**

Among 69,183 patients with advanced PC, 476 underwent conversion surgery; 430 with FOLFIRINOX and 46 with GnP. The median duration from diagnosis to conversion surgery was 6.4 months. Overall survival (OS) was 31.2 months after cancer diagnosis and 23.5 months after conversion surgery. Adjuvant chemotherapy was a significant factor for OS, with hazard ratios (HRs) of 0.23 [95% CI 0.12–0.44, *P* < 0.01] from cancer diagnosis and 0.20 [95% CI 0.10–0.37, *P* < 0.01] from conversion surgery. No significant differences were noted between FOLFIRINOX and GnP. However, maintaining the same regimens as preoperative chemotherapy was a significant factor, with HRs of 0.67 [95% CI 0.47–0.95, *P* = 0.02] from cancer diagnosis and 0.69 [95% CI 0.49–0.98, *P* = 0.04] from conversion surgery.

**Conclusions:**

The incorporation of adjuvant chemotherapy with the same preoperative regimen could be an effective strategy for patients with advanced PC who would undergo conversion surgery.

## Introduction

Conversion surgery refers to a surgical resection performed for tumors that were initially deemed unresectable but have responded to systemic therapy enough to undergo radical resection. At the time of diagnosis, 80% of pancreatic cancers (PCs) are presented with an unresectable advanced stage owing to local invasion or distant metastasis (1). With recent advancements in chemotherapeutic agents, there has been the patients undergoing conversion surgery after systemic chemotherapy even in advanced PC, and the rates have been reported to be approximately 3.6—16% for locally advanced PC and 4.2—7.5% for metastatic PC (2–4). Furthermore, some studies have demonstrated favorable clinical outcomes of conversion surgery (2, 4–17). However, the role of conversion surgery has not yet been established for PC (16–18), although the effectiveness of conversion surgery has been demonstrated particularly for metastatic colon cancer (19, 20).

Regarding chemotherapeutic regimens, contemporary guidelines recommend either 5-fluorouracil, leucovorin/folinic acid, irinotecan, and oxaliplatin (FOLFIRINOX) or gemcitabine with albumin-bound paclitaxel (GnP) as the first-line palliative chemotherapy for unresectable advanced PC. However, it remains unclear which regimen, FOLFIRINOX or GnP, provides superior survival to warrant it being the first-line recommended regimen. Although reports show that FOLFIRINOX results in better survival than GnP, opposite results have been reported (21–23). In a neoadjuvant setting, Hackert T. et al. reported that FOLFIRINOX regimen appears to be the most effective for neoadjuvant therapy on locally advanced PC (24). However, two recently published articles on conversion surgery did not show any survival differences between the two regimens (2, 17). Therefore, we investigated affecting factors related to conversion surgery preoperatively treated with FOLFIRINOX or GnP for patients with advanced PC, including locally advanced and metastatic PC.

## Methods

### Data source

This study was based on National Health Insurance Service (NHIS) data. This national institution that provides healthcare services to people in South Korea, covering almost 97% of all medical conditions except cosmetic treatment (25). Every claim for medical reimbursement is prospectively filed in the NHIS database, which includes extensive information on diagnoses, medications, procedure or surgery codes, and admissions. Furthermore, medical expenses related to rare intractable diseases (RIDs) are supported by a national aid program and a special code (V193) is co-assigned to claims associated with RIDs, including malignant, autoimmune, and inflammatory bowel diseases. The RID code is highly specific to diseases as it can only be assigned after diagnostic code validation by a qualified physician (26). The NHIS database can be accessed for academic purposes from 2010 onward if the request is approved for qualified research. Approval from the Institutional Review Board and Ethics Committee was waived because it did not collect or record personally identifiable information. All procedures were performed in accordance with the principles of the Declaration of Helsinki.

### Establishing the study population

Among the patients who claimed to have PC between 2005 and 2020, only those assigned both PC and V193 codes were eligible for this study. Individuals who underwent conversion surgery after preoperative chemotherapy with FOLFIRINOX or GnP were selected based on the following operational definitions: individuals who claimed chemotherapy reimbursement for at least three consecutive months and subsequently claimed surgical resection. In South Korea, during the study period, the chemotherapeutic regimens FOLFIRINOX and GnP were covered by the NHIS only for patients with either locally advanced or metastatic PC, and not for those with resectable or borderline resectable PC. As a result, when claims are made for FOLFIRINOX or GnP, it can be inferred that the patients are indeed in a locally advanced or metastatic stage of PC.

Regarding the chemotherapy regimen, only individuals treated with FOLFIRINOX or GnP as first-line chemotherapy were included in this study cohort. FOLFIRINOX was defined as the combination of the following drug codes within one reimbursement claim form: fluorouracil, irinotecan, and oxaliplatin. GnP was defined using the drug codes gemcitabine and nab-paclitaxel.

Conversion surgery was defined as the presence of claims for the following surgery codes after preoperative claims for chemotherapy: Q7561 for total pancreatectomy; Q7571 for Whipple’s operation; Q7572 for pylorus-preserving pancreaticoduodenectomy; Q7565 for distal pancreatectomy; Q7567 for spleen preserving distal pancreatectomy; and Q7221, Q7222, and Q7225 for metastatectomy with liver wedge resection.

### Data collection and outcome parameters

Multiple preoperative variables before the date of pancreatic cancer diagnosis were extracted from the claim datasets of the study cohort. Considering the medical history of the underlying diseases, the presence of disease codes of relevant medical conditions, including congestive heart failure, myocardial infarction, cerebrovascular accident, liver cirrhosis, variceal bleeding, diabetes mellitus, chronic renal failure, and previous malignant diseases, except pancreatic cancer, were regarded as having the corresponding illness. The Charlson Comorbidity Index (CCI) was calculated, and the type and timing of conversion surgery, along with preoperative chemotherapy duration, were assessed.

All subjects were followed up from the date of diagnosis of pancreatic cancer until December 31, 2020, or death, whichever occurred first. The primary outcome of this study was overall survival (OS) from the time of conversion surgery, with the aim of mitigating the effect associated with the duration of preoperative chemotherapy. The secondary outcome was recurrence-free survival (RFS). The time of cancer recurrence was defined as follows: 1) for the subset of patients who did not undergo adjuvant chemotherapy after conversion surgery, the date of re-administration of chemotherapy was considered the timing of tumor recurrence; and 2) for the subsets who underwent adjuvant chemotherapy, it was defined as the administration of a new regimen during or after completion of adjuvant chemotherapy. This study was approved by the Institutional Review Board of the National Cancer Center (NCC2021-0091).

### Statistical analyses

Descriptive statistics were used to analyze the baseline characteristics of the patients. Continuous data were reported as mean with standard deviation or median with range. Categorical variables were presented as numbers or proportions. The characteristics and variables of conversion surgeries were compared using a two-sample independent *t*-test for numerical variables and a Pearson chi-square test or Fisher’s exact test for nominal variables. OS and RFS were estimated using the Kaplan-Meier method, and differences among groups were compared using the log-rank test. Statistical analyses were performed using SAS statistical software (version 9.4; SAS Institute, Cary, NC, USA). Statistical significance was set at *P* < 0.05.

## Results

### Flow of study population

**Figure 1:**
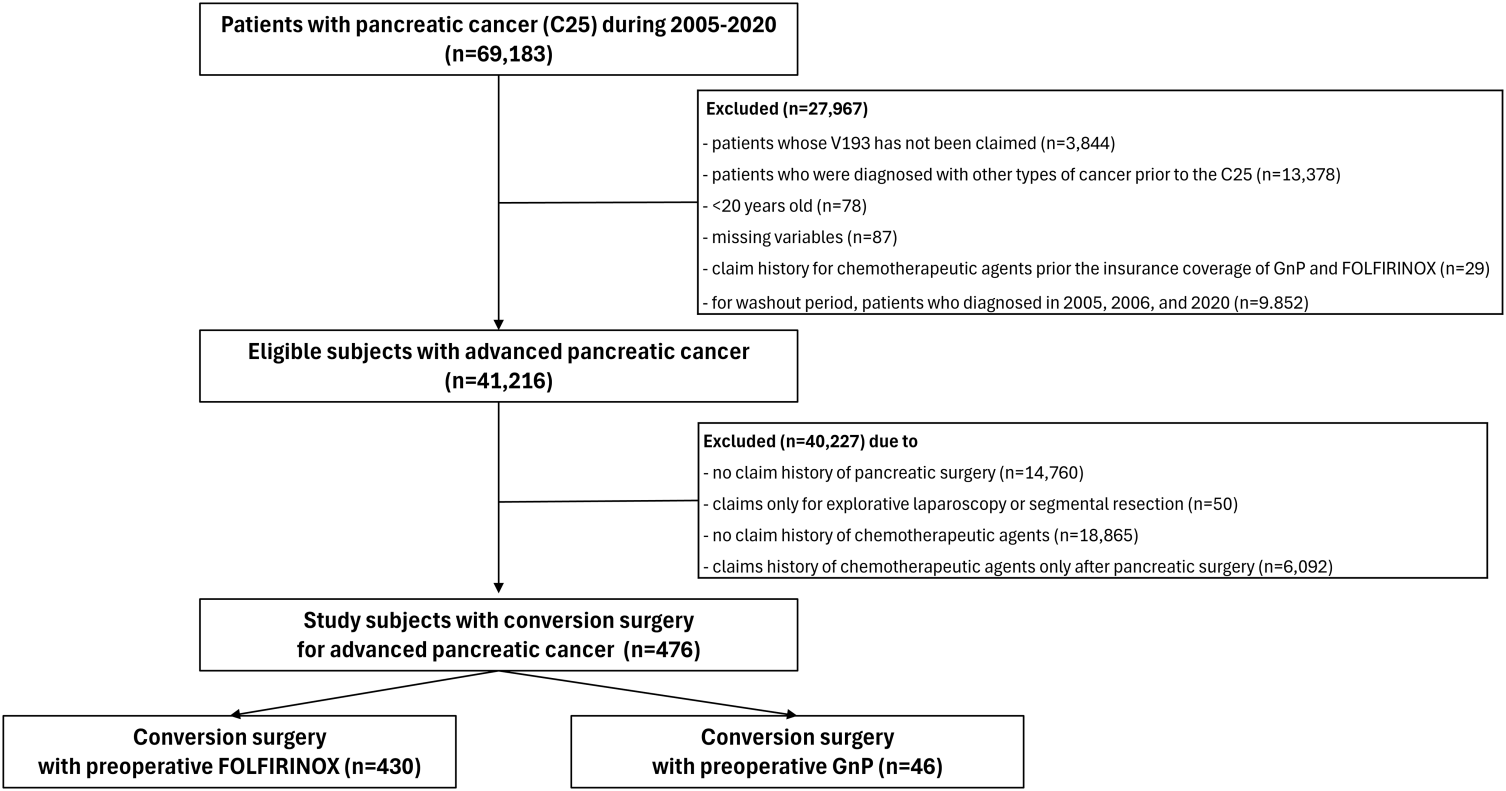
Flow chart of the study population.

In total, 69,183 patients diagnosed with pancreatic cancer between 2005 and 2020 were identified in the NHIS database. Among these, subjects without a V193 code (n=3,844), with a previous history of any cancer (n=13,378) or pancreatic surgery prior to the pancreatic cancer diagnosis (n=699), with missing data for independent variables (n=87), and those younger than 20 years (n=78) were excluded. Patients who were managed with only conservative treatment (n=18,865), palliative chemotherapy (n=14,760), or palliative surgical treatment (explorative laparoscopy, n=510) were excluded. Therefore, a total of 989 patients were treated with preoperative systemic chemotherapy. Finally, only patients treated with FOLFIRINOX or GnP were included in this study population (n=476) and were categorized into two groups (1): conversion surgery after preoperative chemotherapy with FOLFIRINOX (n=430) and (2) conversion surgery after preoperative chemotherapy with GnP (n=46) (**Figure 1**).

### Baseline characteristics

There were no significant differences in the baseline characteristics between the FOLFIRINOX and GnP groups (**Table 1**). The mean age at the time of pancreatic cancer diagnosis was 60.7 ± 9.3 vs. 60.7 ± 10.1 years, with a male proportion of 52.7 and 56.6%, respectively. Furthermore, the proportions of CCI ≤2 were 62.3 vs. 54.3%, and follow-up durations were 23.0 vs. 23.8 months from cancer diagnosis and 16.1 vs. 14.6 months from conversion surgery.

**Table 1:** Baseline characteristics and oncological features related to cancer management.

Variables	Total (n=476)	FOLFIRINOX (n=430)	GnP (n=46)	*P*-value
Age, (mean, SD)	60.9 ± 9.4	60.7 ± 9.3	60.7 ± 10.1	0.98
Male sex, n (%)	253 (53.1)	227 (52.7)	26 (56.6)	0.62
Chalson Comorbidity Index, n (%)				
0-2	293 (61.5)	268 (62.3)	25 (54.3)	0.55
3-6	168 (35.2)	149 (34.6)	19 (41.3)	
≥7	15 (3.1)	13 (3.0)	19 (41.3)	
Duration of preoperative chemotherapy, months (mean with SD), n (%)	6.49 ± 4.8	6.33 ± 4.0	7.9 ± 5.3	0.02
6-8	79 (16.6)	71 (16.5)	8 (17.3)	
<6	293 (61.5)	273 (63.4)	20 (43.4)	
>8	104 (21.8)	86 (20.0)	18 (39.1)	
Follow-up duration, months (median with IQR)				
from the date of cancer diagnosis	23.2 (55.5, 67.0)	23.0 (16.5, 32.6)	23.8 (16.4, 31.0)	0.92
from the date of conversion surgery	16.0 (10.1, 24.6)	16.1 (10.5, 24.6)	14.6 (6.8, 24.0)	0.20
Types of conversion surgery, n (%)				
Whipple's operation	106 (22.27)	101 (23.49)	5 (10.87)	<0.01
pancreatoduodenectomy	197 (41.39)	189 (43.95)	8 (17.39)	
distal pancreatectomy	150 (31.51)	121 (28.14)	29 (63.04)	
spleen preserving distal pancreatectomy	1 (0.21)	0 (0)	1 (2.17)	
total pancreatectomy	22 (4.62)	19 (4.42)	3 (6.52)	
metastectomy (liver wedge resection)	15 (3.15)	10 (2.33)	5 (10.87)	
Radiotherapy, n (%)				
preoperative radiotherapy	86 (18.07)	83 (19.30)	3 (6.52)	0.01
postoperative radiotherapy	94 (19.75)	89 (20.70)	5 (10.87)	
both pre and postoperative radiotherapy	4 (0.84)	4 (0.93)	0 (0)	
Adjuvant chemotherapy after conversion surgery, n (%)	399 (83.82)	360 (83.72)	39 (84.78)	0.85
same regimen with preoperative chemotherapy	172 (36.13)	151 (35.12)	21 (45.65)	0.15
the other regimens	227 (47.69)	209 (48.60)	18 (39.13)	
gemcitabine monotherapy	128 (26.89)	121 (28.14)	7 (15.22)	<0.01
gemcitabine with erlotinib	4 (0.84)	3 (0.70)	1 (2.17)	
5-FU monotherapy	47 (9.87)	45 (10.47)	2 (4.35)	
TS-1	9 (1.89)	6 (1.40)	3 (6.52)	
Gemcitabine with nab-paclitaxel	2 (0.42)	2 (0.47)	–	
FOLFIRINOX	1 (0.21)	–	1 (2.17)	
gemcitabine with cisplatin	1 (0.21)	1 (0.23)	–	
others	29 (6.09)	26 (6.06)	3 (6.52)	
Second-line chemotherapy, n (%)	298 (62.6)	269 (62.5)	29 (63.0)	0.94
gemcitabine monotherapy	148 (31.0)	139 (32.3)	9 (19.5)	<0.01
gemcitabine with erlotinib	9 (1.8)	8 (1.8)	1 (2.1)	
5-FU monotherapy	59 (12.3)	55 (12.7)	4 (8.7)	
TS-1	21 (4.4)	13 (3.0)	8 (17.3)	
Gemcitabine with nab-paclitaxel	3 (0.6)	3 (0.7)	–	
FOLFIRINOX	1 (0.2)	–	1 (2.1)	
gemcitabine with cisplatin	3 (0.6)	3 (0.7)	–	
others	54 (11.3)	48 (11.1)	6 (13.04)	

SD indicates standard deviation; n, number; IQR, interquartile range; FU, fluorouracil; TS-1, tegafur/gimeracil/oteracil potassium.

#### Oncologic and surgical features

The duration of preoperative chemotherapy was significantly longer in the GnP group, while preoperative radiotherapy was higher in the FOLFIRINOX group. However, the proportion of implementing adjuvant chemotherapy after conversion surgery did not differ significantly, with rates of 83.7 and 84.7% for the FOLFIRINOX and GnP, respectively (*P* = 0.85). Regarding the type of adjuvant chemotherapy, the same regimen used for preoperative chemotherapy was most frequently applied in both groups, with rates of 35.1 and 45.6% in the FOLFIRINOX and GnP groups, respectively. However, among the other regimens, except for the preoperative chemotherapy, gemcitabine monotherapy was more frequently selected as adjuvant chemotherapy in the FOLFIRINOX group than in the GnP group. As second-line chemotherapy, 31.0% of the patients were treated most frequently with gemcitabine monotherapy, with rates of 32.3 and 19.5% in the FOLFIRINOX and GnP groups, respectively. In terms of the type of pancreatic surgery, Whipple’s operation and pancreatectomy were more frequently conducted in the FOLFIRINOX, while distal pancreatectomy was most frequently performed in the GnP.

### Survival outcomes

**Figure 2:**
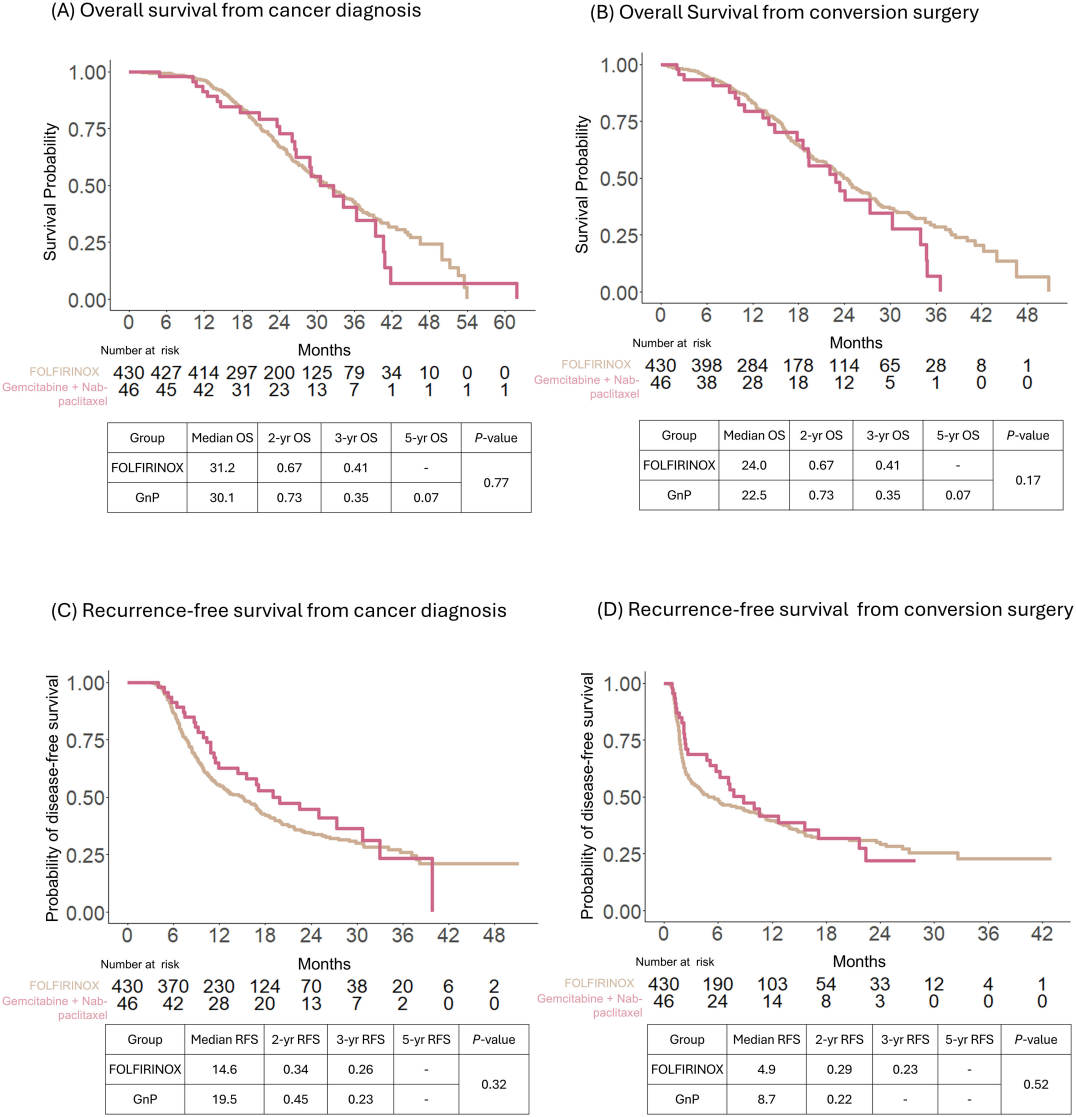
Comparison of survival outcomes of patients with conversion surgery after preoperative treatment with FOLFIRINOX or gemcitabine with nab-paclitaxel. Overall survival (OS) was calculated from the date of cancer diagnosis **(A)** and conversion surgery **(B)**. Recurrence-free survival (RFS) was calculated from the date of cancer diagnosis **(C)** and conversion surgery **(D)**.

The median OS of all patients was 31.2 and 23.5 months after cancer diagnosis and conversion surgery, respectively. The corresponding 3-year survival rates were 41 and 26%, respectively (**Supplementary Figure S1**). In addition, according to the preoperative chemotherapy regimen, the median OS for FOLFIRINOX was 31.2 months from cancer diagnosis and 24.0 months from conversion surgery; the median OS for GnP was 30.1 months from cancer diagnosis and 22.5 months from conversion surgery. The median OS was not significantly different between the FOLFIRINOX and GnP as preoperative chemotherapy preceding conversion surgery (*P* = 0.77 from the date of cancer diagnosis and *P* = 0.17 from the date of conversion surgery) (**Figures 2A, B**). The median RFS for FOLFIRINOX was 14.6 and 4.9 months from cancer diagnosis and conversion surgery, respectively; the median RFS for GnP was19.5 and 8.7 months from cancer diagnosis and conversion surgery, respectively. The median RFS was not significantly different between the FOLFIRINOX and GnP groups (*P* =0.32 from the date of cancer diagnosis and *P* = 0.52 from the date of conversion surgery) (**Figures 2C, D**).

#### Beneficial effects of adjuvant chemotherapy

**Figure 3:**
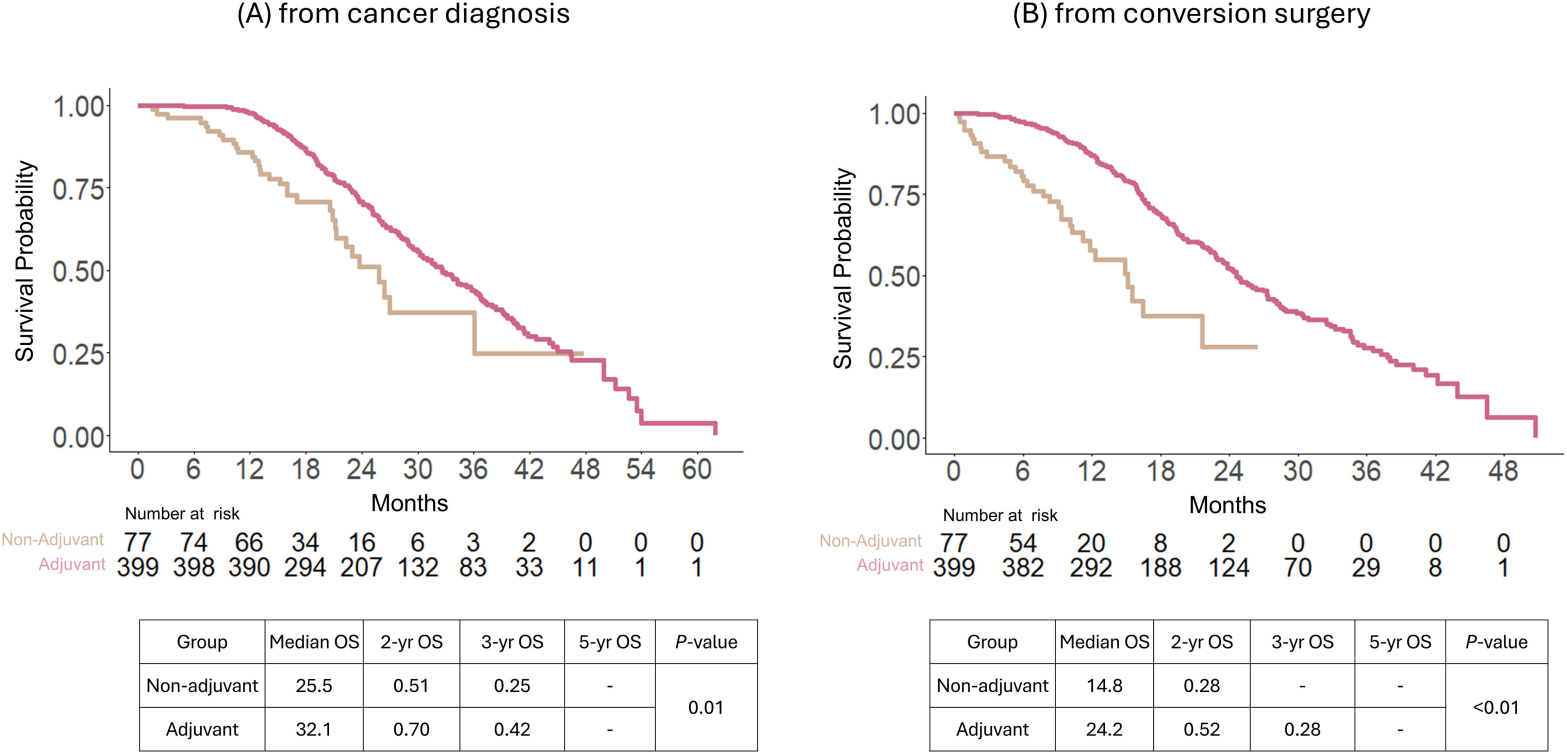
Overall survival comparison between adjuvant and non-adjuvant chemotherapy of patients after conversion surgery with preoperative chemotherapy in the entire study population. Survival analysis was calculated from the date of cancer diagnosis **(A)** and conversion surgery **(B)**.

Even after being treated with preoperative chemotherapy, the implementation of adjuvant chemotherapy after conversion surgery demonstrated survival benefits, as calculated from the dates of both cancer diagnosis and conversion surgery. Between the adjuvant and non-adjuvant groups, the median OS was 32.1 vs. 25.5 months from the date of cancer diagnosis (*P* = 01); 24.2 vs. 14.8 months from the date of conversion surgery (*P* < 0.01). The 2-, 3-, and 5-year OS were 51, 25, and 0% in the non-adjuvant chemotherapy group and 70, 42, and 4 in the adjuvant chemotherapy group from the date of cancer diagnosis, respectively (*P* = 0.01 by log-rank test, **Figure 3A**). Additionally, the 2- and 3-year OS were 28 and 0% in the non-adjuvant chemotherapy group and 52 and 28% in the adjuvant chemotherapy group, respectively, from the date of conversion surgery (*P* < 0.01 by log-rank test; **Figure 3B**).

#### Subset analysis after stratification into the FOLFIRINOX or GnP group

The beneficial effects of adjuvant chemotherapy were identified in a stratified group consisting of only patients preoperatively treated with FOLFIRNIOX (360 with vs. 70 without adjuvant chemotherapy). The median OS calculated was 23.4 months for non-adjuvant vs. 32.1 months for adjuvant chemotherapy (*P* = 0.01) from the date of cancer diagnosis; 14.8 months for non-adjuvant vs. 24.6 months for adjuvant chemotherapy (*P* < 0.01) from the date of conversion surgery (**Supplementary Figure S2**) In the stratified cohort consisting only of patients preoperatively treated with GnP (39 with vs. 7 without adjuvant chemotherapy), the beneficial effects of adjuvant chemotherapy were not statistically significant although the survival graph shows the trends of beneficial effects of adjuvant chemotherapy (**Supplementary Figure S3**).

### Prognostic factors for survival outcomes

Cox regression analyses evaluating prognostic factors are presented in **Table 2** for OS calculated from the date of cancer diagnosis and **Table 3** for OS calculated from the date of conversion surgery. Regarding chemotherapeutic regimens (FOLFIRINOX vs. GnP), OS was not affected by either the method of calculation from the date of cancer diagnosis or conversion surgery. Moreover, radiotherapy did not affect OS in the preoperative or postoperative settings. Based on the date of cancer diagnosis, a longer duration of preoperative chemotherapy was associated with improved OS, whereas based on the date of conversion surgery, it turned out to be statistically insignificant. However, patients who received adjuvant chemotherapy exhibited significantly longer survival than those who did not receive adjuvant chemotherapy after undergoing conversion surgery, with hazard ratios (HRs) of 0.238 [95% CI 0.128–0.442, *P* < 0.01] from the date of cancer diagnosis and 0.201 [95% CI 0.108–0.373, *P* < 0.01] from the date of conversion surgery. Moreover, patients treated with the same regimen of preoperative chemotherapy after conversion surgery showed survival improvement, regardless of whether FOLFIRINOX or GnP was used as preoperative chemotherapy, with HRs of 0.674 [95% CI 0.476–0.956, *P* = 0.02] from the date of cancer diagnosis and 0.698 [95% CI 0.494–0.987, *P* < 0.04] from the date of conversion surgery.

**Table 2:** Factors affecting overall survival from the date of cancer diagnosis.

Variables		Univariate			Multivariate		
		HR	95% CI	*p*-value	HR	95% CI	*p*-value
Age		1.02	1.00-1.03	0.01	1.01	1.00-1.03	0.02
Sex	male vs. female	1.23	0.94-1.59	0.11	1.30	0.99-1.71	0.05
Charlson Comorbidity Index	3≤CCI<7 vs. CCI<3	1.35	1.03-1.77	<0.01	1.26	0.95-1.66	<0.01
	CCI≥7 vs. CCI<3	2.82	1.52-5.23		3.22	1.66-6.23	
Radiotherapy	preoperative RT vs. no RT	0.65	0.44-0.95	0.10	0.74	0.50-1.10	0.39
	postoperative RT vs. no RT	0.86	0.63-1.17		0.85	0.61-1.17	
	both pre- and post-op RT vs. no RT	0.44	0.11-1.81		0.58	0.14-2.41	
First-line CTx	GnP vs. FOLFIRINOX	1.06	0.69-1.63	0.77	0.84	0.54-1.33	0.47
Adjuvant CTx	yes vs. no adjuvant CTx	0.54	0.37-0.80	<0.01	0.23	0.12-0.44	<0.01
Type of adjuvant CTx	same regimen with preop CTx vs. the others	0.98	0.75-1.28	0.90	0.67	0.47-0.95	0.02
Second-line CTx	yes vs. no second line CTx	0.98	0.73-1.31	0.91	1.79	1.14-2.80	0.01
Timing of conversion surgery*	6- <8 mo	ref.		0.01	ref.		0.01
	<6 mo	0.85	0.60-1.20		1.02	0.71-1.46	
	≥8 mo	0.49	0.31-0.75		0.51	0.33-0.79	

*Time from diagnosis to surgery.

CCI indicates Charlson Comorbidity index; RT, radiotherapy; CTx, chemotherapy; mo, months.

**Table 3:** Factors affecting overall survival from the date of conversion surgery.

Variables		Univariate			Multivariate		
		HR	95% CI	*p*-value	HR	95% CI	*p*-value
Age		1.02	1.00-1.03	0.01	1.01	0.99-1.02	0.07
Sex	male vs. female	1.32	1.02-1.72	0.03	1.30	0.99-1.71	0.05
Charlson Comorbidity Index	3≤CCI<7 vs. CCI<3	1.43	1.09-1.87	0.01	1.21	0.92-1.60	0.01
	CCI≥7 vs. CCI<3	2.88	1.55-5.34		3.27	1.69-6.32	
Radiotherapy	preoperative RT vs. no RT	0.79	0.54-1.15	0.25	0.81	0.54-1.19	0.48
	postoperative RT vs. no RT	0.77	0.56-1.05		0.82	0.59-1.14	
	both pre- and post-op RT vs. no RT	0.50	0.12-2.02		0.56	0.13-2.31	
First-line CTx	GnP vs. FOLFIRINOX	1.33	0.88-2.02	0.17	1.01	0.65-1.57	0.94
Adjuvant CTx	yes vs. no adjuvant CTx	0.33	0.22-0.49	<0.01	0.20	0.10-0.37	<0.01
Type of adjuvant CTx	same regimen with preop CTx vs. the others	1.09	0.83-1.43	0.51	0.69	0.49-0.98	0.04
Second-line CTx	yes vs. no second line CTx	0.89	0.67-1.19	0.46	1.79	1.14-2.80	0.01
Timing of conversion surgery*	6- <8 mo	ref.		0.06	ref.		0.52
	<6 mo	0.68	0.48-0.96		0.82	0.57-1.18	
	≥8 mo	0.85	0.55-1.30		0.92	0.60-1.43	

*Time from diagnosis to surgery.

CCI indicates Charlson Comorbidity index; RT, radiotherapy; CTx, chemotherapy; mo, months.

## Discussion

This nationwide population study investigated oncological outcomes and prognostic factors in patients who underwent conversion surgery for advanced PC, including locally advanced or metastatic PC. The median duration from diagnosis to conversion surgery was 6.49 months for all included patients. The median OS was 31.2 months from the date of cancer diagnosis and 23.52 months from the date of conversion surgery. The proportion of patients who received adjuvant chemotherapy after conversion surgery was 83.82%, of whom approximately 35% were treated with the same regimen of preoperative chemotherapy. The incorporation of adjuvant chemotherapy after conversion surgery showed a survival benefit compared to that in the non-adjuvant group. Regarding the chemotherapy regimens, there were no significant differences in OS and RFS between FOLFIRINOX and GnP as preoperative treatments. However, maintaining the same regimens as preoperative treatments was demonstrated to be a significant factor, although the duration of preoperative chemotherapy did not affect survival outcomes. Therefore, the incorporation of adjuvant chemotherapy after conversion surgery with the same preoperative regimen could be an effective strategy for patients who undergo conversion surgery after preoperative systemic chemotherapy for advanced PC, including those with locally advanced and metastatic PC.

Recently, favorable outcomes of conversion surgery after preoperative systemic chemotherapy have been reported especially for the individuals who had exceptionally responded to systemic chemotherapy (2, 4–17). This nationwide study could provide a new evidence on the clinical outcomes of conversion surgery because it has been the largest cohort (n=476) to date from almost the entire Korean population who underwent conversion surgery for advanced PC.

In 2023, the International Association of Pancreatology and Japan Pancreas Society released a position paper on conversion surgery for pancreatic ductal adenocarcinoma with distant abdominal organ metastasis (27). According to the position paper, the indications for conversion surgery are as follows: 1) disappearance of metastatic lesions on imaging studies, 2) maintenance or downsizing of the primary pancreatic tumor to borderline resectability, 3) decreasing tumor markers, and 4) good performance status of the patients (14, 17, 28). Furthermore, the position paper outlines simultaneous pancreatectomy and hepatectomy for pancreatic cancer with synchronous oligo liver metastasis as follows: the liver lesions could be categorized as an oligo metastasis or an occult metastasis without extra-hepatic or distant metastasis by preoperative imaging (6, 8, 29–31). Therefore, the patients treated with systemic chemotherapy should be evaluated for restaging and resectability with a multidisciplinary team, even for metastatic and locally advanced PC, thereby leading to the chance of eligibility for conversion surgery (2, 18, 27, 28). However, the following issues should be addressed to optimize care: 1) the role of adjuvant chemotherapy after conversion surgery, 2) the duration of preoperative chemotherapy and appropriate timing of surgical resection, 3) the role of metastatectomy accompanied by conversion surgery. 4) the justification of conversion surgery in peritoneal carcinomatosis.

This population-based study of 476 patients with advanced pancreatic cancer robustly demonstrated that incorporation of adjuvant chemotherapy after conversion surgery is associated with improved patient’s survival. These results are consistent with those of two recent studies conducted in Asia and Europe. In 2020, a study involving 32 patients (17 with locally advanced PC and 15 with metastatic PC) from the Kansai Medical University in Japan demonstrated that adjuvant chemotherapy was a statistically significant prognostic factor (3). Similarly, a 2022 study of 173 patients with metastatic pancreatic cancer from Heidelberg University Hospital in Germany indicated that adjuvant chemotherapy led to a significant improvement in survival outcomes after conversion surgery (17). However, a study by Nagai et al. from Johns Hopkins Hospital in the U.S. did not show the beneficial effects of adjuvant chemotherapy (31). However, the number of cases was too small to draw a conclusion.

Furthermore, in determining the preferred regimen as preoperative chemotherapy between FOLFIRINOX and GnP, the current study showed no significant differences between the two regimens, which is consistent with previous reports (2, 17). However, this study demonstrated that implementing the same preoperative chemotherapy regimens was a significant factor for survival improvement, regardless of whether FOLFIRINOX or GnP was used as preoperative chemotherapy. It could be plausible given the tumor biology which had exceptionally responded to initial chemotherapy, the responsiveness would be maintained by the same chemotherapy in adjuvant setting after conversion surgery. To the best of our knowledge, this is the first study regarding whether to align with the preoperative chemotherapies or necessitate different regimens. When considering the duration of preoperative chemotherapy before conversion surgery, a longer duration from the date of cancer diagnosis was associated with improved OS. However, based on the date of conversion surgery, it turned out to be statistically insignificant, revealing that the improved survival calculated from the date of cancer diagnosis may be influenced by the duration of preoperative treatment.

This study has some limitations owing to its retrospective design for enrolling patients who underwent conversion surgery. It must be noted that a selection bias can exist because the fact that patients are eligible for conversion surgery implies that they are outstanding responders to systemic chemotherapy, and patients who demonstrated good tolerability for conversion surgery after preoperative chemotherapy, without postoperative deterioration in physical status or complications, would be more likely to be treated with the same preoperative chemotherapy regim. Otherwise, patients might be treated with a less toxic chemotherapy regimen or managed conservatively to avoid toxicity. Moreover, we could not differentiate between locally advanced and metastatic PC stages. Therefore, we focused on the prognostic factors within individuals who successfully underwent conversion surgery and further evaluated prognostic factors based on survival, which was calculated from the date of conversion surgery as well as cancer diagnosis, thereby mitigating the effect of different cancer stages. Finally, cancer-specific clinical features such as metastatic status (number and extent of distant metastases), surgical outcomes (completeness of resection, complications), pathologic responsiveness (margin status, pathologic findings, and lymph nodes) and serum levels of the tumor marker CA 19-9 could not be evaluated in this study, although these are well-known prognostic factors for favorable outcomes of conversion surgery (17, 19, 20).

## Conclusion

In conclusion, the incorporation of adjuvant chemotherapy with the same preoperative regimen could be an effective strategy for patients with advanced PC who are good responders to systemic chemotherapy and undergo conversion surgery.

## Data Availability

Publicly available datasets were analyzed in this study. This data can be found here: Retrospective Cohort of the National Health Insurance System in South Korea (https://nhiss.nhis.or.kr).

## Supplementary material

Supplementary Figure 1 Overall survival of patients who underwent conversion surgery for advanced pancreatic cancer in the entire study population. Survival analysis was calculated from the date of cancer diagnosis **(A)** and conversion surgery **(B)**.

Supplementary Figure 2 Subset analysis of overall survival between adjuvant and non-adjuvant chemotherapy of patients after stratification into the FOLFIRINOX group. Survival analysis was calculated from the date of cancer diagnosis **(A)** and conversion surgery **(B)**.

Supplementary Figure 3 Subset analysis of overall survival between adjuvant and non-adjuvant chemotherapy of patients after stratification into the gemcitabine with nab-paclitaxel group. Survival analysis was calculated from the date of cancer diagnosis **(A)** and conversion surgery **(B)**.
