# Supplementary figures and images for: Survival benefits of adjuvant chemotherapy after conversion surgery in patients with advanced pancreatic cancer
Source: Front Oncol. 2025 Jan 7;14:1510016. doi: 10.3389/fonc.2024.1510016 (PMC11746061; doi:10.3389/fonc.2024.1510016)

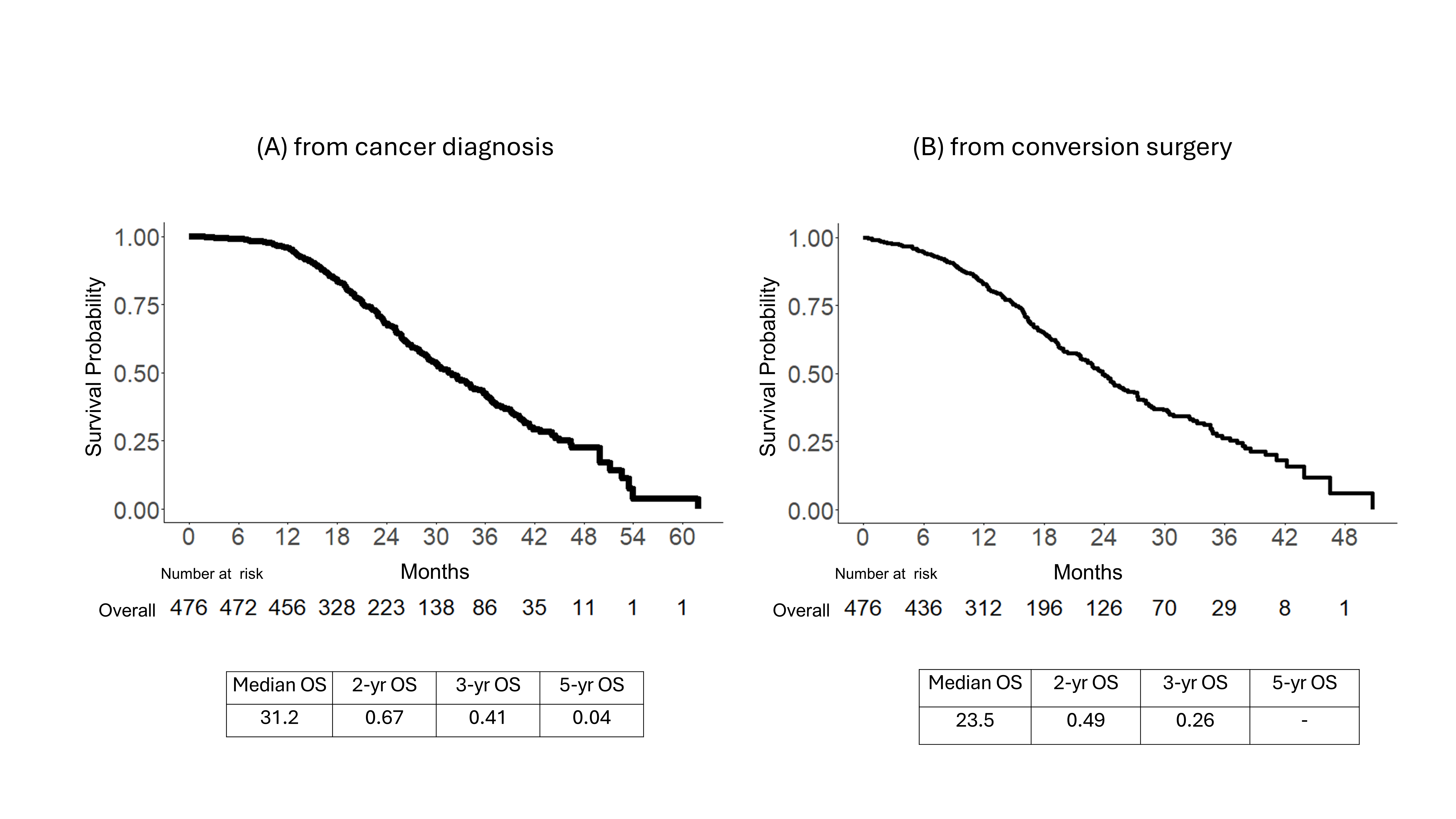

Supplement: Supplementary Figure 1 — Overall survival of patients who underwent conversion surgery for advanced pancreatic cancer in the entire study population. Survival analysis was calculated from the date of cancer diagnosis (A) and conversion surgery (B). [file Image1.tif]

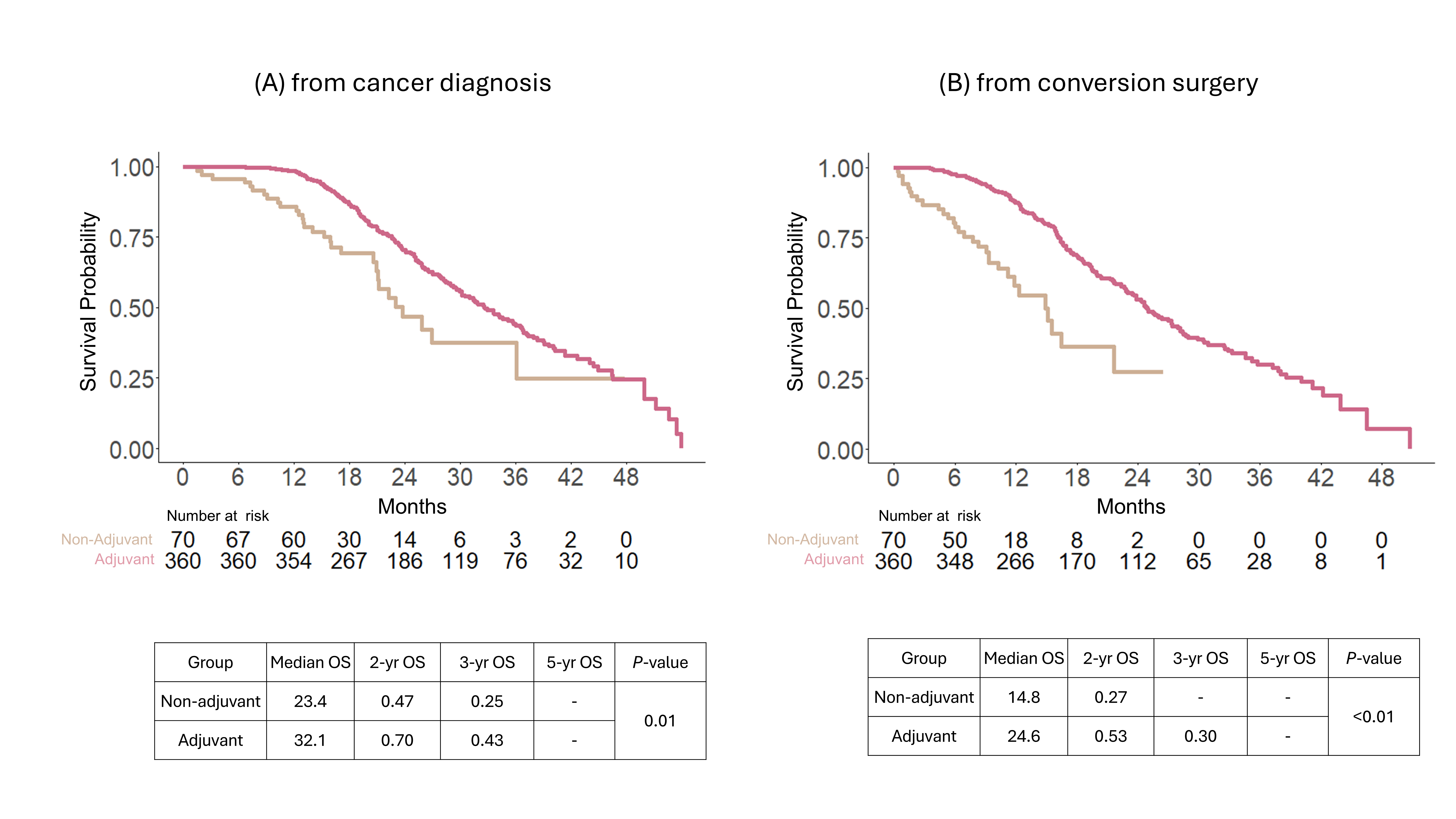

Supplement: Supplementary Figure 2 — Subset analysis of overall survival between adjuvant and non-adjuvant chemotherapy of patients after stratification into the FOLFIRINOX group. Survival analysis was calculated from the date of cancer diagnosis (A) and conversion surgery (B). [file Image2.tif]

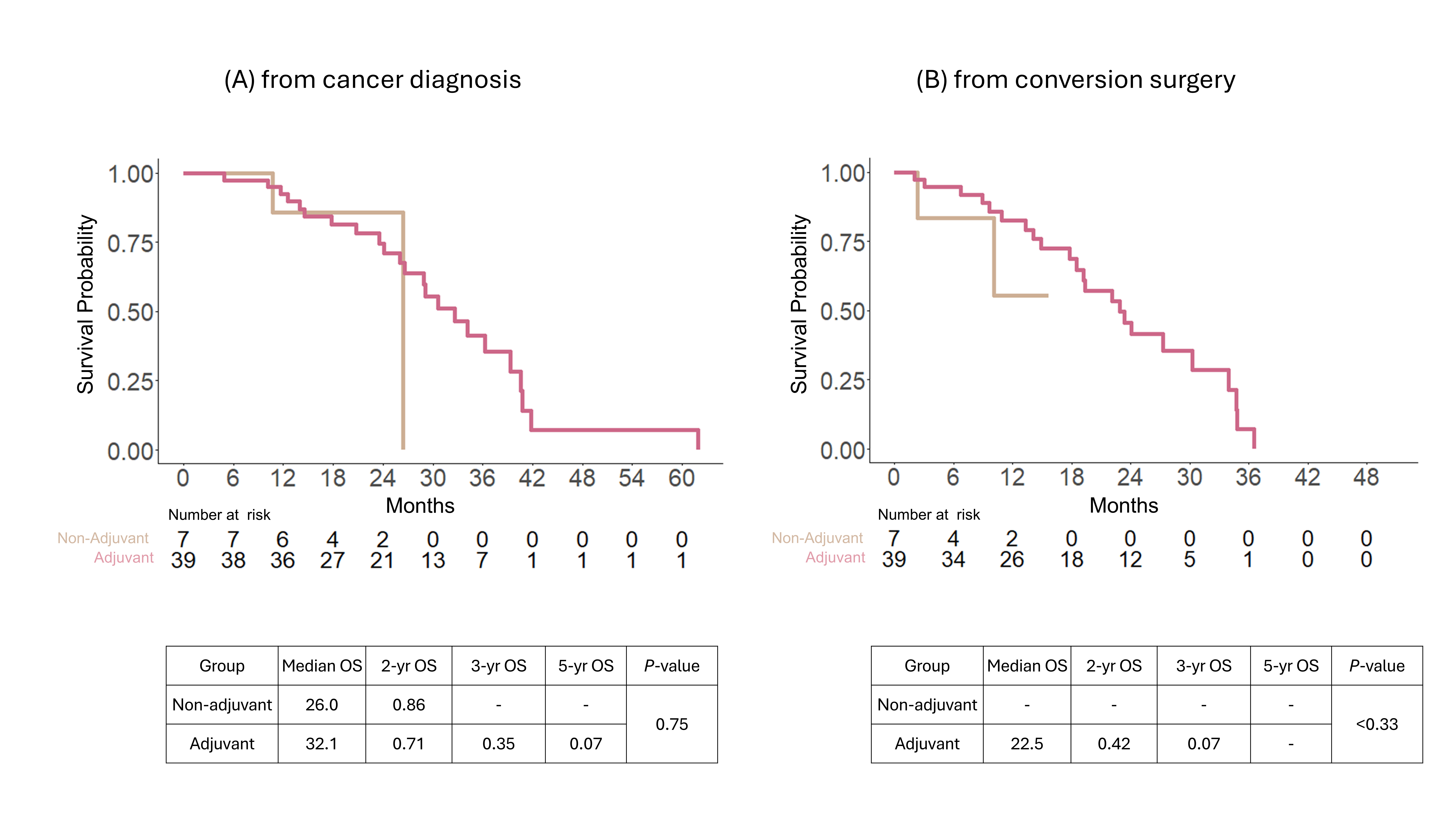

Supplement: Supplementary Figure 3 — Subset analysis of overall survival between adjuvant and non-adjuvant chemotherapy of patients after stratification into the gemcitabine with nab-paclitaxel group. Survival analysis was calculated from the date of cancer diagnosis (A) and conversion surgery (B). [file Image3.tif]
